# 1546. Relationship between Measured Glomerular Filtration Rate and Tenofovir Dried Blood Spot Concentrations among Transgender Adults on Tenofovir Alafenamide/Emtricitabine for Pre-Exposure Prophylaxis

**DOI:** 10.1093/ofid/ofad500.1381

**Published:** 2023-11-27

**Authors:** Nimish Patel, Linda Awdishu, Leah Burke, Sheldon Morris

**Affiliations:** University of California San Diego, San Diego, California; University of California San Diego, San Diego, California; University of California San Diego, San Diego, California; University of California San Diego, San Diego, California

## Abstract

**Background:**

Kidney function can alter drug clearance. This is pertinent for tenofovir-based pre-exposure prophylaxis (PrEP) regimens where drug concentrations confer protection against HIV. Among transgender (TG) individuals whose kidney function is dynamic due to use of gender affirming therapy (GAT), it is unclear if there is a relationship between tenofovir diphosphate dried blood spots (TFV-DP DBS) and direct measures of glomerular filtration rate (mGFR) The objective of this study was to quantify this relationship and compare if mGFR differs between TG persons with perfect/imperfect adherence to tenofovir alafenamide/emtricitabine (TAF/FTC).

**Methods:**

A prospective cohort study was performed among TG individuals on TAF/FTC for PrEP. Inclusion criteria were age ≥18 years old, TG identity, HIV negative, use of PrEP for ≥12 weeks, and willing to receive iohexol for direct measurement of GFR. TFV DBS concentrations were evaluated after being on ≥12 weeks of TAF/FTC. Perfect adherence to TAF/FTC was defined as a TFV-DP DBS of >1800fmol/punch (Yager et al, PMID: 32539288). Iohexol clearance was used for mGFR by subcutaneously administering 0.5ml iohexol. Plasma iohexol concentrations were assayed 3h post-injection. Pearson’s correlation coefficients were computed to quantify the relationship between TFV-DP DBS and iohexol clearance. Median iohexol clearances were compared between those with perfect/imperfect adherence.

**Results:**

Among the 28 individuals included, median (interquartile range, IQR) age was 33.0 (26.3 – 36.8) years. Three quarters were on GAT and over two thirds (67.9%) were assigned male at birth (AMAB). Median (interquartile range, IQR) iohexol clearance and TFV-DP DBS concentrations were 76 (65 – 95)ml/min and 2611 (2114 – 3240), respectively. There was no significant correlation between TFV-DP DBS and iohexol clearance (r^2^=0.10, 95% confidence interval, CI: -0.29 – 0.45, p=0.62). TFV-DP DBS >1800fmol/punch (perfect adherence) was observed in 22 participants. Median (95% CI) iohexol clearance between those with and without perfect adherence did not reach significance: 78 (68-96) vs 69 (60-83)mL/min, respectively (p=0.26).

**Conclusion:**

No significant relationship was observed between iohexol clearance and TFV-DP DBS among TG individuals taking TAF/FTC for PrEP.

**Disclosures:**

**Sheldon Morris, MD**, Gilead: Grant/Research Support

